# Association of dietary intake of saturated fatty acids with hypertension: 1999–2018 National Health and Nutrition Examination Survey

**DOI:** 10.3389/fnut.2022.1006247

**Published:** 2022-11-03

**Authors:** Ruoyu Gou, Yufan Gou, Jian Qin, Tingyu Luo, Qiannan Gou, Kailian He, Song Xiao, Ruiying Li, Tingjun Li, Jie Xiao, Ziqi Chen, Yulu Chen, You Li, Zhiyong Zhang

**Affiliations:** ^1^Guangxi Key Laboratory of Environmental Exposomics and Entire Lifecycle Heath, Department of Environmental Health and Occupational Medicine, School of Public Health, Guilin Medical University, Guilin, China; ^2^Department of Political Science and Administration, School of International Affairs and Public Administration, Ocean University of China, Qingdao, China; ^3^Department of Environmental and Occupational Health, School of Public Health, Guangxi Medical University, Nanning, China; ^4^Department of Food Science, Faculty of Veterinary and Agricultural, The University of Melbourne, Parkville, VIC, Australia

**Keywords:** dietary saturated fatty acids, subtype, cardiovascular disease, dose-response, NHANES

## Abstract

**Objective:**

This study aimed to assess the relationship between the dietary intake of saturated fatty acids (SFAs) and its subtypes (C4:0, C6:0, C8:0, C10:0, C12:0, C14:0, C16:0, and C18:0) and hypertension.

**Design, participants, and methods:**

Adults aged 20 years and older based used the U.S. Health and Nutrition Survey (1999–2018) were used as participants. Two averages of 24 h dietary recall data were obtained for weight-adjusted continuous cross-sectional analysis. Two 24-h recall interview data means were obtained for weight-adjusted continuous cross-sectional analysis. A logistic regression model was used to estimate the weighted odds ratio (OR) and its 95% confidence interval (CI) for hypertension.

**Results:**

The study included 7,222 respondents over 20 years of age with a hypertension prevalence of 23.2% and a significant difference in the dietary intake of carbohydrates among patients with hypertension. Dietary intake of nutrients was more in men than in women with hypertension. After adjusting for confounders, adjusting for nutrients, and reducing covariance among nutrients, the OR (95% CI) for women’s dietary intake of SFAs, C14:0, C16:0, C18:0 fourth quartile, and C14:0 third quartile were 0.57 (0.34, 0.95), 0.57 (0.34, 0.95), 0.57 (0.34, 0.95), 0.57 (0.34, 0.95), and 0.57 (0.34, 0.95), respectively, which may be a risk factor for hypertension. In older (≥65, years) respondents, the OR (95% CI) for dietary intake of SFAs, C4:0, C14:0, C16:0 fourth quartile, and C12:0 third quartile were 0.42 (0.21, 0.86), 0.46 (0.22, 0.95), 0.39 (0.18, 0.85), 0.38 (0.17, 0.84), and 0.45 (0.20, 0.99), respectively, which may be a protective factor for hypertension.

**Conclusion:**

The study was based on the American Health and Nutrition Examination Survey, and a strong correlation was found between dietary intake of SFAs, C14:0, C16:0, and C18:0 and hypertension in women (dietary intake of SFAs, C4:0, C12:0, C14:0, and C16:0) and middle-aged and older adults (dietary intake of SFAs, C4:0, C12:0, C14:0, and C16:0). In addition, dietary nutrient intake should be carefully selected for the rational prevention of hypertension.

## Introduction

According to the World Health Organization, 17 million people die each year from cardiovascular diseases. Hypertension accounts for 45% of all heart disease-related mortality. Approximately 40% of adults aged 25 years and older are diagnosed with hypertension worldwide, and the prevalence of hypertension has reached 35% among adults in the United States (1, 2). Hypertension has become a global public health problem that cannot be ignored.

Common factors influencing hypertension include dietary, psychosocial, socioeconomic status, and genetic (3). Diet plays an important role in cardiovascular disease risk, and adherence to a scientific diet is an essential strategy to reduce the incidence of hypertension. The protective effect of nutrients on hypertension is not negligible (4, 5). Among them, saturated fatty acids (SFAs), polyunsaturated fatty acids, and monounsaturated fatty acids are closely related to hypertension (6, 7). Common sources of SFA are coconut oil, rice bran oil, red meat, high-fat dairy products, and human breast milk. In general, SFAs are considered to be a risk factor for hypertension (8). The Dietary Guidelines for Americans recommend reducing SFA to less than 7% of energy to reduce the incidence of cardiovascular diseases (9). However, one study has found that SFA may improve cardiovascular health (7).

Studies have reported that the smaller the chain length, the stronger the effect of raising HDL (10). Gender (11) and age have a significant effect on patients with hypertension. High levels of SFA in serum correlate with hypertension in men (12). Dietary intake of SFAs, C12:0, C14:0, and C16:0 in the elderly is beneficial to health (7). A study found that the effect of SFA intake on hypertension was not significant in young and middle-aged adults compared with older adults. Therefore, the present study investigated the relationship between SFAs and their subtypes and hypertension, which may provide some reference for the dietary intake of SFAs and subtypes to reduce the occurrence of hypertension.

## Materials and methods

### Study design and population

**FIGURE 1:**
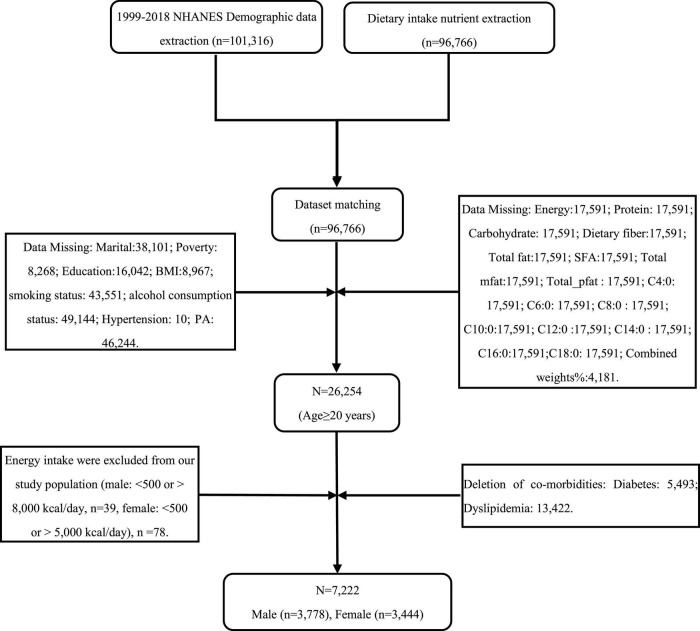
Flow chart of the participants’ selection.

Based on the publicly available data from the National Health and Nutrition Examination Survey (NHANES) (13, 14), trained professional investigators conducted face-to-face, telephone interviews, and laboratory tests to collect data. The National Center for Health Statistics Institutional Review Board/Ethics Review Board approved this survey (NHANES, 1999–2016)^1^ to obtain informed consent from participants and continue the study through home interviews and examinations. This study used a case-control study to obtain 10 cross-sectional survey cycles of NHANES database respondents (*n* = 7,222) between 1999 and 2018. The study population was estimated to be representative of the United States (*n* = 38,123,806), the hypertension group (*n* = 5,550), and the control group (*n* = 1,672). Complete questionnaire data, laboratory test data, reliable data of two 24 h recalled dietary intakes, and accurate calculation of nutrient intake were obtained. Exclusion criteria were as follows: (1) respondents aged < 20 years; (2) excluding energy intake (male: <500 or >8,000 kcal/day, female: <500 or >5,000 kcal/day); (3) respondents with diabetes, hyperlipidemia; (4) repeat respondents; (5) respondents with missing data (Figure 1).

### Assessment of nutrients

Dietary sources of energy, protein, carbohydrate, dietary fiber, total fat, dietary monounsaturated fatty acids, polyunsaturated fatty acids, SFAs, and their subtypes (C4:0, C6:0, C8:0, C10:0, C12:0, C14:0, C16:0, and C18:0) were obtained by two 24-h recall interviews. Nutrient types were selected and calculated with reference to NHANES support material^2^. The instrument for measuring the dietary intake of respondents was described in detail in the measurement guide (15). Dietary nutrient intakes were calculated using the Food and Nutrition Database for Dietary Studies 5.0 (FNDDS 5.0) standard of the Ministry of Agriculture (16, 17).

### Definition of hypertension

Participants’ blood pressure measurements were taken according to the NHANES description (18), with systolic blood pressure (SBP) and diastolic blood pressure (DBP) taken as the average of three or more measurements. Hypertension is defined as (1) taking hypertensive medication; (2) being informed by a licensed physician of hypertension or stating in the questionnaire to take prescribed medication for hypertension; and (3) measuring the participant’s SBP mean ≥ 140 mmHg and/or DBP ≥ 90 mmHg (mean of three times) (19). Any one of the above three conditions can be diagnosed as hypertension (20).

### Measurements of covariates

Demographic information was collected by questionnaire, including age (20–44, 45–64, 65, and older), gender, race (non-Hispanic white, non-Hispanic black, Hispanic, and others), smoking status (never smoked [less than 100 cigarettes in a lifetime], formerly smoked [smoked more than 100 cigarettes in lifetime, now not smoking at all], and currently smoking [smoked more than 100 cigarettes in lifetime, some days, or every day]), education (less than high school, high school graduate/general education, some college, or above), and family income to poverty ratio (less than 1.30, 1.30 to <3.00, 3.00 to <5.00, 5.00 and above) represents the ratio of family income to the federal poverty threshold, adjusting for household size. A higher ratio indicates higher levels of income, marital status (marriage, divorce, unmarried), and drinking status (current heavy drinker [women drink ≥ 3 drinks per day/men drink ≥ 4 drinks per day/binge drinking 5 days or more]; current moderate drinkers [women drink ≥ 2 drinks per day/men drink ≥ 3 drinks per day/binge drinking ≥ 2 days per month]; current light alcohol users [not the above two categories] (6); and no alcohol consumption). Physical activity (using total Mets for the week: <600, Mets, min/week, when exercise ≥ 600, Mets, min/week. Exercise also has significant physical benefits (21). The diagnosis of diabetes consists of the following components, any of which can be met to be diagnosed with diabetes: (1) the individual has been diagnosed with diabetes by a doctor; (2) glycosylated hemoglobin (HbA1c) (%) > 6.5; (3) fasting blood glucose (DM, mmol/l) ≥ 7.0; (4) random blood glucose (mmol/L) ≥ 11.1; (5) 2 h OGTT blood glucose (mmol/l) ≥ 11.1; and (6) use of diabetes medication or insulin. Body mass index (BMI) was as follows: <25 kg/m^2^, 25–30 kg/m^2^, and ≥30 kg/m^2^. Hyperlipidemia was diagnosed by meeting any of the following criteria: hypertriglyceridemia (TG ≥ 150 mg/dL) and/or hypercholesterolemia {TG 200 mg/dL [5.18 mmol/L], LDL 130 mg/dL [3.37 mmol/L], HDL < 40 mg/dL [1.04 mmol/L] (male), 50 mg/L [1.30mmol/L] (female)}.

## Statistical analysis

Continuous variables were described by mean ± SD, and continuous variables were compared using Student’s *t*-test. Categorical variables were described by *n* (%), and they were compared between groups using a chi-squared test. Nutrient intake was corrected using the residual method. Nutrient intakes were grouped into quartiles and analyzed for the inter-quartile group trend. The lowest quartile (first quartile) was defined as the reference group in each model. The results were expressed as weighted (OR [95% CI]). Nutrients entered into the model using a variance inflation factor (<10) (22) (energy, kcal: 42.763; protein, g: 3.127; carbohydrate, g: 13.381; dietary fiber, g: 1.977, total fat, g: 44.032; total mfat, g: 17.812; total pfat, g: 6.083). Logistic regression models were used to analyze the association between SFAs and their subtypes (C4:0, C6:0, C8:0, C10:0, C12:0, C14:0, C16:0, and C18:0) and hypertension. Stratified analyses and interactions were performed in accordance with sex, age, and BMI to explore the effects of SFAs and their subtypes on hypertension in different exposure settings. All analyses were performed in R (4.1.3) language, using NHANESR (version 0.9.2.8) package, survey (version 4.1-1) package compareGroups (version 4.5.2) package, forest plot (version 2.0.1) package, etc. for statistical analysis. A two-tailed *P*-value of < 0.05 was considered statistically significant.

## Results

Table 1 shows the number of respondents and the weighted percentages. In the total population with 10 survey periods, a significant difference in age and BMI (*P-value* < 0.001) was observed between the hypertension group and control group, with the highest prevalence of hypertension (45–64, years). The prevalence was higher in men than in women in the total population. Gender and age are recognized risk factors for hypertension, which will be explored in a stratified analysis with interaction.

**TABLE 1:** Socio-demographic characteristics of US adults with hypertension by NHANES survey cycle, 1999–2018 (*N* = 7,222).

Parameter	No. of participants (weighted %)^a^											
	All participants			1999–2000			2001–2002			2003–2004		
	Non-HTN (*n* = 5,550)	HTN (*n* = 1,672)	*P-value*	Non-HTN (*n* = 360)	HTN (*n* = 131)	*P-value*	Non-HTN (*n* = 526)	HTN (*n* = 188)	*P-value*	Non-HTN (*n* = 503)	HTN (*n* = 181)	*P-value*
**Age group**												
20–44, years	4273 (77.53)	567 (38.25)	<0.001	268 (81.24)	44 (52.05)	<0.001	386 (76.81)	57 (40.50)	<0.001	393 (80.66)	48 (33.34)	<0.001
45–64, years	1035 (19.52)	662 (42.59)		68 (16.20)	36 (24.15)		119 (21.19)	78 (43.10)		84 (17.33)	70 (43.32)	
≥65, years	242 (2.95)	443 (19.16)		24 (2.56)	51 (23.80)		21 (2.00)	53 (16.40)		26 (2.01)	63 (23.34)	
**Gender**												
Male	2824 (49.37)	954 (57.05)	<0.001	202 (50.47)	78 (58.03)	0.200	268 (50.38)	95 (49.45)	0.900	270 (48.32)	91 (50.73)	0.680
Female	2726 (50.63)	718 (42.95)		158 (49.53)	53 (41.97)		258 (49.62)	93 (50.55)		233 (51.68)	90 (49.27)	
**Race/ethnicity**												
White	2653 (70.67)	744 (69.38)	<0.001	187 (76.52)	69 (72.73)	0.200	317 (78.38)	98 (75.00)	0.010	281 (74.22)	104 (74.95)	0.280
Black	1194 (10.82)	561 (17.60)		66 (8.73)	29 (14.57)		95 (8.80)	61 (17.30)		114 (11.28)	46 (14.87)	
Mexican	722 (6.76)	170 (5.01)		73 (5.05)	24 (4.73)		78 (5.60)	19 (3.32)		68 (6.05)	19 (2.88)	
Other	981 (11.75)	197 (8.01)		34 (9.70)	9 (7.97)		36 (7.22)	10 (4.38)		40 (8.45)	12 (7.30)	
**Marital**												
Married	3021 (56.43)	979 (63.21)	<0.001	210 (60.24)	81 (60.62)	0.100	321 (62.18)	103 (59.52)	0.003	268 (55.68)	117 (68.14)	0.003
Separated	628 (10.21)	404 (20.66)		40 (11.71)	36 (21.73)		61 (9.58)	51 (24.06)		58 (10.88)	44 (20.99)	
Never married	1901 (33.36)	289 (16.13)		110 (28.05)	14 (17.65)		144 (28.24)	34 (16.42)		177 (33.44)	20 (10.87)	
**Ratio of family income** **to poverty level**^b^												
<1.3	1464 (19.40)	475 (21.44)	0.350	91 (18.73)	40 (27.27)	0.270	93 (13.45)	48 (23.92)	0.010	115 (16.87)	40 (16.42)	0.670
1.3–3	1673 (26.53)	524 (27.33)		98 (22.42)	37 (23.60)		150 (24.43)	57 (22.31)		173 (32.58)	63 (27.35)	
3–5	1246 (26.92)	345 (24.13)		87 (27.55)	27 (23.87)		149 (32.49)	35 (18.04)		108 (23.77)	37 (26.08)	
≥5	1167 (27.15)	328 (27.10)		84 (31.30)	27 (25.26)		134 (29.63)	48 (35.73)		107 (26.78)	41 (30.15)	
**Education levels**												
Less than 11th grade	820 (9.59)	364 (14.62)	<0.001	90 (14.63)	47 (28.81)	0.030	79 (9.98)	51 (18.07)	0.030	84 (9.92)	38 (10.20)	0.740
High school graduate	1136 (20.18)	386 (22.24)		75 (20.99)	29 (23.96)		130 (22.71)	42 (20.30)		103 (21.56)	50 (24.86)	
College graduate or above	3594 (70.23)	922 (63.14)		195 (64.38)	55 (47.23)		317 (67.31)	95 (61.63)		316 (68.52)	93 (64.94)	
**BMI**												
<25, kg/m^2^	2964 (56.09)	510 (29.83)	<0.001	225 (63.93)	41 (28.58)	<0.001	304 (62.12)	80 (41.34)	0.001	285 (59.34)	59 (29.52)	<0.001
25–30, kg/m^2^	1568 (27.20)	567 (35.26)		90 (25.07)	42 (33.17)		148 (24.16)	57 (27.67)		132 (25.46)	61 (34.63)	
≥30, kg/m^2^	1018 (16.81)	595 (34.91)		45 (11.00)	48 (38.25)		74 (13.72)	51 (30.99)		86 (15.20)	61 (35.85)	
**Smoking consumption status**												
Never	3435 (61.26)	811 (46.75)	<0.001	211 (58.69)	66 (50.26)	0.060	287 (54.35)	88 (43.43)	0.120	289 (57.31)	91 (53.44)	0.010
Former	908 (18.28)	465 (30.15)		58 (15.23)	43 (27.43)		110 (22.72)	41 (23.50)		74 (16.10)	52 (29.04)	
Now	1207 (20.46)	396 (23.10)		91 (26.08)	22 (22.31)		129 (22.93)	59 (33.07)		140 (26.59)	38 (17.52)	
**PA**												
<600, Mets, min/week	1675 (30.38)	579 (34.75)	0.020	224 (61.92)	79 (62.77)	0.870	317 (57.97)	120 (65.44)	0.160	325 (63.80)	100 (54.19)	0.110
≥600, Mets, min/week	3875 (69.62)	1093 (65.25)		136 (38.08)	52 (37.23)		209 (42.03)	68 (34.56)		178 (36.20)	81 (45.81)	
**Alcohol consumption status**												
Never	603 (9.41)	193 (8.67)	<0.001	35 (7.04)	18 (8.75)	0.010	57 (11.95)	21 (11.94)	0.300	47 (7.87)	24 (9.70)	
Former	476 (7.44)	274 (13.56)		35 (8.79)	29 (19.63)		54 (9.26)	32 (15.61)		45 (7.03)	42 (19.72)	<0.001
Mild	1890 (34.56)	621 (38.53)		124 (33.52)	48 (41.10)		201 (39.91)	66 (34.90)		173 (33.45)	70 (40.68)	
Moderate	1120 (21.32)	258 (17.30)		68 (23.60)	14 (9.95)		93 (16.17)	33 (19.85)		101 (21.82)	20 (15.41)	
Heavy	1461 (27.27)	326 (21.94)		98 (27.05)	22 (20.57)		121 (22.71)	36 (17.70)		137 (29.83)	25 (14.49)	

**Parameter**	**No. of Participants (Weighted%)^a^**											
	**2005–2006**			**2007–2008**			**2009–2010**			**2011–2012**		
	**Non-HTN** **(*n* = 573)**	**HTN** **(*n* = 169)**	***P-value***	**Non-HTN** **(*n* = 524)**	**HTN** **(*n* = 156)**	***P-value***	**Non-HTN** **(*n* = 625)**	**HTN** **(*n* = 158)**	***P-value***	**Non-HTN** **(*n* = 640)**	**HTN** **(*n* = 192)**	***P-value***

**Age group**												
20–44, years	438 (77.13)	70 (39.36)	<0.001	401 (79.17)	50 (37.82)	<0.001	492 (76.98)	43 (32.74)	<0.001	515 (76.06)	67 (37.27)	<0.001
45–64, years	104 (19.46)	57 (44.22)		92 (17.23)	70 (45.77)		111 (20.69)	66 (41.79)		106 (21.90)	77 (42.17)	
≥65, years	31 (3.41)	42 (16.42)		31 (3.60)	36 (16.41)		22 (2.33)	49 (25.47)		19 (2.04)	48 (20.56)	
**Gender**												
Male	292 (48.86)	92 (47.67)	0.830	276 (45.85)	95 (56.71)	0.090	295 (47.62)	86 (59.03)	0.070	347 (50.85)	117 (59.71)	0.130
Female	281 (51.14)	77 (52.33)		248 (54.15)	61 (43.29)		330 (52.38)	72 (40.97)		293 (49.15)	75 (40.29)	
**Race/ethnicity**												
White	312 (74.81)	79 (74.97)	0.020	263 (71.61)	78 (73.85)	0.230	332 (72.44)	76 (70.65)	0.060	244 (66.98)	74 (68.54)	0.180
Black	133 (10.06)	65 (17.90)		117 (11.67)	48 (15.12)		98 (9.51)	52 (17.93)		150 (11.01)	75 (17.26)	
Mexican	81 (5.63)	20 (5.31)		75 (7.71)	14 (4.25)		86 (5.60)	16 (5.72)		58 (8.15)	10 (4.76)	
Other	47 (9.50)	5 (1.82)		69 (9.01)	16 (6.78)		109 (12.45)	14 (5.70)		188 (13.86)	33 (9.44)	
Marital												
Married	335 (61.29)	106 (66.36)	0.002	285 (53.28)	98 (72.35)	<0.001	329 (52.88)	89 (67.60)	<0.001	296 (49.83)	111 (61.16)	0.010
Separated	71 (11.63)	37 (20.60)		63 (9.97)	35 (16.36)		75 (10.22)	42 (16.89)		68 (10.84)	40 (17.92)	
Never married	167 (27.08)	26 (13.04)		176 (36.75)	23 (11.29)		221 (36.90)	27 (15.51)		276 (39.33)	41 (20.92)	
**Ratio of family income** **to poverty levels**^b^												
<1.3	122 (14.08)	34 (13.46)	0.950	145 (20.59)	40 (17.98)	0.890	191 (19.17)	45 (21.51)	0.710	210 (27.74)	60 (22.94)	0.530
1.3–3	173 (25.48)	53 (24.25)		167 (27.01)	49 (27.03)		188 (26.13)	46 (25.00)		178 (25.45)	63 (33.49)	
3–5	151 (34.04)	47 (33.75)		108 (24.87)	34 (24.26)		129 (26.13)	41 (29.53)		124 (24.35)	32 (21.91)	
≥5	127 (26.40	35 (28.4		104 (27.53)	33 (30.73)		117 (28.57)	26 (23.96)		128 (22.46)	37 (21.66)	
**Education levels**												
Less than 11th grade	84 (8.52)	40 (15.78)	0.120	106 (14.87)	37 (16.30)	0.620	95 (9.83)	17 (6.56)	0.360	81 (10.00)	46 (17.51)	0.070
High school graduate	118 (19.58)	33 (21.38)		110 (21.30)	32 (17.61)		127 (17.99)	43 (24.32)		103 (15.90)	43 (20.96)	
College graduate or above	371 (71.90	96 (62.84)		308 (63.83)	87 (66.10)		403 (72.18)	98 (69.11)		456 (74.10)	103 (61.53)	
**BMI**												
<25, kg/m^2^	302 (59.08)	47 (36.26)	0.003	277 (53.50)	42 (27.29)	<0.001	338 (57.94)	48 (24.53)	<0.001	332 (52.97)	54 (30.26)	<0.001
25–30, kg/m^2^	163 (25.86)	66 (37.80)		159 (28.61)	64 (40.35)		181 (27.70)	47 (32.24)		189 (29.31)	74 (43.76)	
≥30, kg/m^2^	108 (15.06)	56 (25.94)		88 (17.89)	50 (32.37)		106 (14.36)	63 (43.23)		119 (17.72)	64 (25.98)	
**Smoking consumption status**												
Never	337 (60.09)	86 (43.20)	0.050	318 (59.63)	70 (48.02)		379 (59.41)	77 (52.61)	0.190	426 (61.13)	96 (43.25)	0.001
Former	102 (17.60)	46 (29.57)		84 (17.84)	48 (30.82)	0.080	107 (21.39)	46 (29.08)		92 (17.53)	58 (40.10)	
Now	134 (22.31	37 (27.23)		122 (22.53)	38 (21.16)		139 (19.19)	35 (18.32)		122 (21.34)	38 (16.65)	
**PA**												
<600, Mets, min/week	346 (60.18)	104 (61.49)	0.850	76 (14.76)	29 (17.46)	0.570	94 (15.05)	30 (14.59)	0.900	85 (11.47)	25 (11.07)	0.890
≥600, Mets, min/week	227 (39.82)	65 (38.51)		448 (85.24)	127 (82.54)		531 (84.95)	128 (85.41)		555 (88.53)	167 (88.93)	
**Alcohol consumption status**												
Never	60 (9.25)	19 (7.21)	0.170	52 (9.35)	20 (9.04)	0.110	64 (9.30)	17 (9.48)	0.010	77 (8.42	24 (10.42)	0.550
Former	64 (9.99)	39 (19.36)		64 (9.15)	33 (13.39)		56 (8.32)	31 (22.29)		54 (8.08)	26 (10.58)	
Mild	179 (33.86)	53 (32.85)		152 (30.85)	59 (44.15)		196 (33.50)	59 (36.33		208 (31.69)	66 (35.66)	
Moderate	106 (19.93)	29 (17.17)		104 (20.22)	16 (11.72)		118 (19.75)	19 (12.46)		129 (20.05)	39 (22.14)	
Heavy	164 (26.97)	29 (23.41)		152 (30.43)	28 (21.71)		191 (29.13)	32 (19.44)		172 (31.76)	37 (21.20)	

**Parameter**		**No. of participants (weighted%)^a^**										
		**2013–2014**				**2015–2016**				**2017–2018**		
		**Non-HTN** **(*n* = 679)**	**HTN** **(*n* = 192)**		***P-value***	**Non-HTN** **(*n* = 607)**	**HTN** **(*n* = 157)**		***P-value***	**Non-HTN** **(*n* = 513)**	**HTN** **(*n* = 148)**	***P-value***

**Age group**												
20–44, years		510 (71.55)	76 (39.98)		<0.001	470 (77.67)	55 (35.12)		<0.001	400 (79.98)	57 (36.34)	<0.001
45–64, years		144 (24.68)	72 (41.12)			109 (17.60)	69 (49.89)			98 (17.29)	67 (48.40)	
≥65, years		25 (3.76)	44 (18.90)			28 (4.73)	33 (14.99)			15 (2.73)	24 (15.26)	
**Gender**									
Male		356 (51.74)	122 (63.81)		0.070	289 (48.76)	89 (58.39)		0.230	229 (50.14)	89 (68.20)	0.040
Female		323 (48.26)	70 (36.19)			318 (51.24)	68 (41.61)			284 (49.86)	59 (31.80)	
**Race/ethnicity**									
White		301 (64.77)	74 (61.35)		0.010	246 (67.69)	40 (54.33)		0.020	170 (62.92)	52 (65.85)	0.110
Black		138 (11.43)	64 (20.17)			146 (12.46)	59 (22.06)			137 (12.43)	62 (18.94)	
Mexican		75 (9.01)	20 (7.78			71 (7.13)	19 (8.03)			57 (6.97)	9 (4.10)	
Other		165 (14.79)	34 (10.70)			144 (12.72)	39 (15.58)			149 (17.68)	25 (11.11)	
**Marital**									
Married		366 (55.84)	110 (58.54)		0.002	340 (62.49)	80 (58.05)		<0.001	271 (51.11)	84 (61.12)	0.030
Separated		82 (10.94)	42 (20.66)			54 (6.71)	44 (24.93)			56 (10.29)	33 (22.03)	
Never married		231 (33.22)	40 (20.80)			213 (30.80)	33 (17.02)			186 (38.60)	31 (16.85)	
**Ratio of family income** **to poverty levels**^b^									
<1.3		205 (22.92)	64 (25.67)		0.230	162 (21.82)	58 (27.13)		0.550	130 (17.60)	46 (20.13)	0.850
1.3–3		165 (22.22)	63 (29.30)			211 (32.49)	51 (32.63)			170 (26.32)	42 (27.20)	
3–5		157 (25.57)	34 (18.11)			118 (20.53)	25 (13.29)			115 (30.15)	33 (31.96)	
≥5		152 (29.29)	31 (26.92)			116 (25.16)	23 (26.95)			98 (25.93)	27 (20.71)	
**Education levels**									
Less than 11th grade		82 (10.08)	39 (16.89)		0.040	67 (6.58)	31 (11.32)		0.130	52 (4.35)	18 (6.04)	0.730
High school graduate		133 (18.28)	47 (23.64)			126 (18.95)	34 (15.85)			111 (25.17)	33 (27.27)	
College graduate or above		464 (71.65)	106 (59.47)			414 (74.47)	92 (72.83)			350 (70.48)	97 (66.69)	
**BMI**									
<25, kg/m^2^		354 (51.76)	68 (34.34)		0.004	290 (49.39)	43 (20.18)		<0.001	257 (53.38)	28 (20.69)	0.010
25–30, kg/m^2^		203 (30.63)	62 (34.69)			165 (28.05)	53 (37.44)			138 (26.21)	41 (30.74)	
≥30, kg/m^2^		122 (17.61)	62 (30.96)			152 (22.56)	61 (42.38)			118 (20.41)	79 (48.57)	
**Smoking consumption status**									
Never		430 (62.19)	85 (45.12)		0.070	411 (68.78)	75 (44.56)		0.003	347 (68.44)	77 (45.01)	0.010
Former		111 (20.20)	57 (29.33)			89 (15.30)	40 (34.84)			81 (18.14)	34 (27.31)	
Now		138 (17.60)	50 (25.55)			107 (15.91)	42 (20.60)			85 (13.42)	37 (27.68)	
**PA**									
<600, Mets, min/week		87 (14.39)	39 (23.44)		0.030	74 (11.57)	27 (11.54)		0.990	47 (6.12)	26 (17.55)	0.030
≥600, Mets, min/week		592 (85.61)	153 (76.56)			533 (88.43)	130 (88.46)			466 (93.88)	122 (82.45)	
**Alcohol consumption status**									
Never		75 (10.68)	18 (7.05)		0.600	82 (11.81)	16 (7.16)		0.370	54 (7.53)	16 (5.52	0.500
Former		57 (7.36)	22 (6.86)			47 (7.58)	20 (10.80)			0 (0)	0 (0)	
Mild		238 (32.12)	76 (38.77)			212 (33.86)	48 (35.61)			207 (41.82)	76 (46.10)	
Moderate		139 (22.45)	36 (23.42)			131 (23.59)	25 (20.70)			131 (25.38)	27 (15.60)	
Heavy		170 (27.38)	40 (23.90)			135 (23.16)	48 (25.73)			121 (25.27)	29 (32.78)	

HTN, hypertension; non-HTN, non-hypertension; BMI, body mass index; PA, physical activity; NHANES, National Health and Nutrition Examination Survey.

^a^Percentages were adjusted for NHANES survey weights.

^b^Represents the ratio of family income to the federal poverty threshold, adjusting for household size. The higher the ratio, the higher the income level.

Tables 2, 3 shows the description of demographic characteristics and nutrient intake. The ratio of family income to poverty levels did not differ significantly between genders. In the hypertension group, no significant differences in race and level of education were observed between genders. In the control group, age and PA level did not differ significantly between genders. All other demographic indicators showed statistically significant differences. Carbohydrate versus C10:0 in the total population was significantly different in the hypertensive group versus the control group. Nutrient intake was lower in women than in men.

**TABLE 2:** Participant characteristics by hypertension status with sex differences: a cross-sectional study using NHANES data from 1999 to 2018.

Parameter	All participants (*n* = 7,222)			HTN group (*n* = 1,672)			Non-HTN group (*n* = 5,550)		
	Male (*n* = 3,778)	Female (*n* = 3,444)	*P*-value	Male (*n* = 954)	Female (*n* = 718)	*P*-value	Male (*n* = 2,824)	Female (*n* = 2,726)	*P*-value
**Age group**									
20–44, years	4273 (77.52	567 (38.25)	<0.001	345 (42.72)	222 (32.32)	0.010	2150 (77.82	2123 (77.24)	0.770
45–64, years	1035 (19.53)	662 (42.59)		344 (38.61)	318 (47.86)		524 (19.09)	511 (19.96)	
≥65, years	242 (2.95)	443 (19.16)		265 (18.67)	178 (19.82)		150 (3.09)	92 (2.80)	
**Race/ethnicity**									
White	2653 (70.67)	744 (69.38)	<0.001	431 (69.17)	313 (69.66)	0.320	1286 (67.83)	1367 (73.44)	<0.001
Black	1194 (10.81	561 (17.60)		308 (16.59)	253 (18.94)		678 (11.96)	516 (9.71)	
Mexican	722 (6.77)	170 (5.01)		98 (5.56)	72 (4.28)		390 (7.85)	332 (5.71)	
Other	981 (11.75)	197 (8.01)		117 (8.68)	80 (7.12)		470 (12.37)	511 (11.14)	
Marital									
Married	3021 (56.43)	979 (63.21)	<0.001	586 (64.82)	393 (61.06)	<0.001	1495 (52.78)	1526 (59.98)	<0.001
Separated	628 (10.21)	404 (20.66)		185 (14.88)	219 (28.35)		253 (7.88)	375 (12.49)	
Never married	1901 (33.36)	289 (16.13)		183 (20.30)	106 (10.59)		1076 (39.34)	825 (27.53)	
**Ratio of family income** **to poverty levels**^b^									
<1.3	1464 (19.40)	475 (21.44)	0.350	266 (21.60)	209 (21.24)	0.760	773 (19.87)	691 (18.94)	0.250
1.3–3	1673 (26.53)	524 (27.33)		305 (27.59)	219 (26.99)		883 (27.62)	790 (25.47)	
3–5	1246 (26.92)	345 (24.12)		202 (22.80)	143 (25.88)		594 (25.53)	652 (28.27)	
≥5	1167 (27.15)	328 (27.11)		181 (28.01)	147 (25.89)		574 (26.98)	593 (27.32)	
**Education levels**									
Less than 11th grade	820 (9.59)	364 (14.62	< 0.001	226 (14.71)	138 (14.51)	0.740	514 (12.15)	306 (7.09)	<0.001
High school graduate	1136 (20.18)	386 (22.24)		226 (23.05)	160 (21.16)		683 (24.23)	453 (16.24)	
College graduate or above	3594 (70.23)	922 (63.14)		502 (62.24)	420 (64.33)		1627 (63.62)	1967 (76.67)	
**BMI**									
<25, kg/m^2^	2964 (56.00)	510 (29.83)	<0.001	278 (25.82)	232 (35.16)	0.004	1438 (50.71)	1526 (61.16)	<0.001
25–30, kg/m^2^	1568 (27.19)	567 (35.26)		352 (39.15)	215 (30.07)		916 (32.06)	652 (22.45)	
≥30, kg/m^2^	1018 (16.81)	595 (34.91)		324 (35.03)	271 (34.77)		470 (17.23)	548 (16.39)	
**Smoking consumption status**									
Never	908 (18.28)	465 (30.15)	<0.001	304 (33.59)	161 (25.57)	0.010	507 (20.40)	401 (16.22)	< 0.001
Former	3435 (61.26)	811 (46.75)		392 (42.01)	419 (53.04)		1538 (54.75)	1897 (67.61)	
Now	1207 (20.46)	396 (23.10)		258 (24.40)	138 (21.39)		779 (24.85)	428 (16.17)	
**PA**									
<600, Mets, min/week	1675 (30.38)	579 (34.75)	0.020	279 (28.06)	300 (43.64)	< 0.001	816 (29.37)	859 (31.37)	0.220
≥600, Mets, min/week	3875 (69.62)	1093 (65.25)		675 (71.94)	418 (56.36)		2008 (70.63)	1867 (68.63)	
**Alcohol consumption status**									
Never	476 (7.44)	274 (13.56)	< 0.001	159 (12.81)	115 (14.57)	< 0.001	271 (8.49)	205 (6.42)	< 0.001
Former	1461 (27.27)	326 (21.94)		209 (23.93)	117 (19.30)		820 (29.99)	641 (24.62)	
Mild	1890 (34.56)	621 (38.53)		392 (43.24)	229 (32.27)		1103 (39.27)	787 (29.97)	
Moderate	1120 (21.31)	258 (17.30)		119 (13.97)	139 (21.71)		407 (14.77)	713 (27.68)	
Heavy	603 (9.42)	193 (8.67)		75 (6.05)	118 (12.15)		223 (7.48)	380 (11.31)	

^b^Represents the ratio of family income to the federal poverty threshold, adjusting for household size. The higher the ratio, the higher the income level.

**TABLE 3:** Characteristics of the weighted dietary intake of nutrients in the hypertensive and non-hypertensive groups among the participants^a^.

Parameter	All participants (*n* = 7,222)	Non-HTN group (*n* = 5,550)	HTN group (*n* = 1,672)	*P-value*	Non-HTN group		*P-value*	HTN group		*P-value*
					Male (*n* = 2,824)	Female (*n* = 2,726)		Male (*n* = 954)	Female (*n* = 718)	
Energy, kcal	2238.78 ± 14.80	2247.76 ± 15.82	2200.36 ± 28.60	0.120	2619.39 ± 26.49	1885.38 ± 17.00	<0.001	2463.28 ± 36.10	1851.11 ± 32.64	<0.001
Protein, g	86.84 ± 0.61	87.12 ± 0.65	85.63 ± 1.26	0.260	102.30 ± 0.99	72.32 ± 0.73	<0.001	95.88 ± 1.69	72.02 ± 1.41	<0.001
Carbohydrate, g	269.23 ± 2.01	271.70 ± 2.19	258.68 ± 3.68	0.001	313.70 ± 3.76	230.75 ± 2.39	<0.001	286.45 ± 5.20	221.79 ± 3.86	<0.001
Dietary fiber, g	17.63 ± 0.18	17.72 ± 0.20	17.25 ± 0.32	0.160	19.43 ± 0.31	16.05 ± 0.25	<0.001	18.66 ± 0.44	15.38 ± 0.37	<0.001
Total fat, g	85.00 ± 0.70	84.84 ± 0.75	85.68 ± 1.40	0.570	98.08 ± 1.25	71.94 ± 0.80	<0.001	94.64 ± 1.86	73.78 ± 2.03	<0.001
Total mfat, g	30.75 ± 0.28	30.61 ± 0.30	31.34 ± 0.58	0.250	35.56 ± 0.51	25.79 ± 0.29	<0.001	34.96 ± 0.83	26.52 ± 0.74	<0.001
Total pfat, g	19.01 ± 0.18	18.98 ± 0.19	19.14 ± 0.36	0.660	21.39 ± 0.33	16.62 ± 0.23	<0.001	20.80 ± 0.37	16.95 ± 0.67	<0.001
SFAs^b^, g	27.62 ± 0.26	27.63 ± 0.28	27.58 ± 0.51	0.920	32.24 ± 0.45	23.13 ± 0.30	<0.001	30.41 ± 0.71	23.81 ± 0.65	<0.001
C4:0, g	0.57 ± 0.01	0.58 ± 0.01	0.55 ± 0.02	0.090	0.66 ± 0.02	0.50 ± 0.01	<0.001	0.58 ± 0.02	0.50 ± 0.02	0.010
C6:0, g	0.32 ± 0.00	0.32 ± 0.01	0.31 ± 0.01	0.100	0.37 ± 0.01	0.28 ± 0.01	<0.001	0.33 ± 0.01	0.28 ± 0.01	0.001
C8:0, g	0.26 ± 0.00	0.26 ± 0.00	0.25 ± 0.01	0.100	0.29 ± 0.01	0.23 ± 0.01	<0.001	0.27 ± 0.01	0.23 ± 0.01	0.001
C10:0, g	0.50 ± 0.01	0.50 ± 0.01	0.47 ± 0.01	0.040	0.56 ± 0.01	0.44 ± 0.01	<0.001	0.51 ± 0.02	0.42 ± 0.02	<0.001
C12:0, g	0.81 ± 0.02	0.82 ± 0.02	0.81 ± 0.03	0.840	0.88 ± 0.02	0.75 ± 0.02	<0.001	0.84 ± 0.04	0.76 ± 0.05	0.001
C14:0, g	2.35 ± 0.03	2.36 ± 0.03	2.29 ± 0.06	0.210	2.74 ± 0.05	2.00 ± 0.04	<0.001	2.48 ± 0.08	2.03 ± 0.07	<0.001
C16:0, g	14.99 ± 0.13	14.99 ± 0.15	15.01 ± 0.26	0.940	17.58 ± 0.23	12.46 ± 0.16	<0.001	16.64 ± 0.38	12.84 ± 0.33	<0.001
C18:0, g	6.86 ± 0.07	6.84 ± 0.08	6.93 ± 0.13	0.530	8.04 ± 0.12	5.67 ± 0.07	<0.001	7.66 ± 0.19	5.95 ± 0.16	<0.001

^a^Mean ± SD was detected by Student’s *t*-test.

^b^SFAs is the sum of C4:0, C6:0, C8:0, C10:0, C12:0, C14:0, C16:0, and C18:0.

Total mfat, total monounsaturated fatty acids; total pfat, total polyunsaturated fatty acids.

Table 4 demonstrates the relationship between SFAs and their subtypes and hypertension. No significant correlation was found between SFAs and their subtypes and hypertension.

**TABLE 4:** Univariate logistic regression results of the relationship between saturated fatty acids and their subtypes and hypertension.

Subgroups	*T-value*	*P-value*	OR (95% CI)
**SFAs**			
[–42.786, –4.638] (*n* = 1,806)	Ref		
[–4.638, –0.128] (*n* = 1,805)	0.91	0.37	1.12 (0.87, 1.44)
[–0.128, 4.571] (*n* = 1,805)	1.43	0.15	1.19 (0.94,1.50)
[4.571, 116.405] (*n* = 1,806)	0.87	0.38	1.11 (0.88, 1.41)
**C4:0**			
[–1.565, –0.259] (*n* = 1,798)	Ref		
[–0.259, –0.077] (*n* = 1, 814)	–1.43	0.16	0.86 (0.70, 1.06)
[–0.077, 0.15] (*n* = 1,806)	–1.37	0.17	0.85 (0.68, 1.07)
[0.15, 7.977] (*n* = 1,804)	–1.80	0.07	0.82 (0.66, 1.02)
**C6:0**			
[–0.873, –0.145] (*n* = 1,803)	Ref		
[–0.145, –0.041] (n = 1,804)	–1.38	0.17	0.87 (0.70, 1.06)
[–0.041, 0.086] (n = 1,805)	–0.16	0.88	0.98 (0.76, 1.27)
[0.086, 4.301] (*n* = 1,810)	–1.94	0.05	0.81 (0.65, 1.00)
**C8:0**			
[–0.681, –0.106] (*n* = 1,796)	Ref		
[–0.106, –0.026] (*n* = 1,804)	0.61	0.55	1.07 (0.86, 1.34)
[–0.026, 0.074] (*n* = 1,821)	0.24	0.81	1.03 (0.81, 1.30)
[0.074, 3.4787] (*n* = 1,801)	–0.38	0.71	0.96 (0.76, 1.20)
**C10:0**			
[–1.291, –0.183] (*n* = 1,811)	Ref		
[–0.183, –0.035] (*n* = 1,800)	0.45	0.65	1.06 (0.83, 1.33)
[–0.035, 0.15] (*n* = 1,802)	–0.28	0.78	0.96 (0.75, 1.24)
[0.15, 5.596] (*n* = 1,809)	–1.19	0.24	0.87 (0.69, 1.10)
**C12:0**			
[–1.916, –0.315] (*n* = 1,809)	Ref		
[–0.315, –0.103] (*n* = 1,797)	0.51	0.61	1.06 (0.84, 1.35)
[–0.103, 0.209] (*n* = 1,808)	–0.34	0.74	0.96 (0.77, 1.20)
[0.209, 22.855] (*n* = 1,808)	–0.44	0.66	0.96 (0.78, 1.17)
**C14:0**			
[–5.382, –0.819] (*n* = 1,806)	Ref		
[–0.819, –0.2] (*n* = 1,805)	–0.26	0.79	0.97 (0.78, 1.21)
[–0.2, 0.518] (*n* = 1,806)	–0.18	0.86	0.98 (0.77, 1.25)
[0.518, 23.636] (*n* = 1,805)	–1.20	0.23	0.87 (0.70, 1.09)
**C16:0**			
[–22.230, –2.123] (*n* = 1,806)	Ref		
[–2.123, 0.161] (*n* = 1,806)	0.29	0.77	1.03 (0.82, 1.31)
[0.161, 2.444] (*n* = 1,804)	2.09	0.04	1.27 (1.01, 1.59)
[2.444, 46.1789] (*n* = 1,806)	1.25	0.21	1.17 (0.91, 1.49)
**C18:0**			
[–10.703, –1.313] (*n* = 1,805)			
[–1.313, –0.125] (*n* = 1,806)	2.54	0.01	1.34 (1.07, 1.67)
[–0.125, 1.073] (*n* = 1,804)	1.72	0.09	1.25 (0.97, 1.63)
[1.073, 17.2571] (*n* = 1,807)	2.43	0.02	1.35 (1.06, 1.71)

Multivariate logistic regressio*n* models adjusted for covariates (gender, age, race, marital, ratio of family income to poverty levels, education level, BMI, smoking consumption status, alcohol consumption status, PA, protein, dietary fiber, total polyunsaturated fatty acids [total pfat]).

In Table 5, no association was found between the dietary intake of SFAs and their subtypes and hypertension by gender. In Table 5, dietary intake of SFAs and their subtypes were not found to be associated with hypertension in different age groups. Inter-quartile trend analysis indicated a linear relationship between the C18:0 and C4:0 quartile subgroups of dietary intake.

**TABLE 5:** Weighted odds ratio sum (95% CI) of quartiles of hypertension and adjusted dietary saturated fatty acid intake: a cross-sectional study using NHANES 1999–2018 data (*N* = 7,222).

Subgroups	Model I^a^		Model II^b^		Model III^c^		
	*P-value*	OR (95% CI)	*P-value*	OR (95% CI)	*P-value*	OR (95% CI)	*P-trend* ^d^
**SFAs**							
[–42.786, –4.638] (*n* = 1,806)	Ref						0.172
[–4.638, –0.128] (*n* = 1,805)	0.91	0.91 (0.59, 1.41)	0.90	0.90 (0.58, 1.39)	0.88	0.88 (0.57, 1.36)	
[–0.128, 4.571] (*n* = 1,805)	0.85	0.85 (0.47, 1.54)	0.85	0.85 (0.47, 1.55)	0.85	0.85 (0.47, 1.56)	
[4.571, 116.405] (*n* = 1,806)	0.97	0.97 (0.46, 2.05)	0.97	0.97 (0.47, 2.01)	0.98	0.98 (0.47, 2.05)	
**C4:0**							
[–1.565, –0.259] (*n* = 1,798)	Ref						0.046
[–0.259, –0.077] (*n* = 1,814)	0.73	0.73 (0.52, 1.02)	0.77	0.77 (0.55, 1.07)	0.77	0.77 (0.55, 1.07)	
[–0.077, 0.15] (*n* = 1,806)	0.73	0.73 (0.50, 1.07)	0.76	0.76 (0.51, 1.12)	0.76	0.76 (0.51, 1.12)	
[0.15, 7.977] (*n* = 1,804)	0.84	0.84 (0.50, 1.42)	0.92	0.92 (0.53, 1.60)	0.91	0.91 (0.53, 1.58)	
**C6:0**							
[–0.873, –0.145] (*n* = 1,803)	Ref						0.487
[–0.145, –0.041] (*n* = 1,804)	0.83	0.83 (0.53, 1.28)	0.83	0.83 (0.53, 1.31)	0.83	0.83 (0.53, 1.29)	
[–0.041,0.086] (*n* = 1,805)	0.93	0.93 (0.54, 1.60)	0.94	0.94 (0.54, 1.64)	0.92	0.92 (0.53, 1.59)	
[0.086, 4.301] (*n* = 1,810)	0.64	0.64 (0.33, 1.21)	0.62	0.62 (0.32, 1.21)	0.61	0.61 (0.32, 1.18)	
**C8:0**							
[–0.681, –0.106] (*n* = 1,796)	Ref						0.169
[–0.106, –0.026] (*n* = 1,804)	1.25	1.25 (0.78, 2.01)	1.28	1.28 (0.78, 2.10)	1.28	1.28 (0.78, 2.08)	
[–0.026, 0.074] (*n* = 1,821)	1.62	1.62 (0.86, 3.03)	1.76	1.76 (0.91, 3.44)	1.78	1.78 (0.92, 3.46)	
[0.074, 3.4787] (*n* = 1,801)	1.91	1.91 (0.92, 3.96)	1.9	1.90 (0.90, 4.02)	1.93	1.93 (0.92, 4.08)	
**C10:0**							
[–1.291, –0.183] (*n* = 1,811)	Ref						0.07
[–0.183, –0.035] (*n* = 1,800)	1.06	1.06 (0.63, 1.79)	1.03	1.03 (0.59, 1.79)	1.06	1.06 (0.61, 1.86)	
[–0.035, 0.15] (*n* = 1,802)	0.85	0.85 (0.44, 1.65)	0.86	0.86 (0.43, 1.70)	0.90	0.90 (0.45, 1.80)	
[0.15, 5.596] (*n* = 1,809)	0.75	0.75 (0.34, 1.65)	0.79	0.79 (0.34, 1.83)	0.84	0.84 (0.36, 1.96)	
**C12:0**							
[–1.916, –0.315] (*n* = 1,809)	Ref						0.199
[–0.315, –0.103] (*n* = 1,797)	0.98	0.98 (0.69, 1.38)	0.94	0.94 (0.67, 1.33)	0.94	0.94 (0.67, 1.33)	
[–0.103, 0.209] (*n* = 1,808)	0.79	0.79 (0.55, 1.15)	0.76	0.76 (0.53, 1.09)	0.76	0.76 (0.53, 1.10)	
[0.209, 22.855] (*n* = 1,808)	0.79	0.79 (0.51, 1.23)	0.77	0.77 (0.49, 1.19)	0.76	0.76 (0.49, 1.19)	
**C14:0**							
[–5.382, –0.819] [*n* = 1,806)	Ref						0.088
[–0.819, –0.2] (*n* = 1,805)	1.02	1.02 (0.69, 1.51)	0.96	0.96 (0.64, 1.43)	0.92	0.92 (0.61, 1.38)	
[–0.2, 0.518] (*n* = 1,806)	1.17	1.17 (0.71, 1.92)	1.08	1.08 (0.64, 1.84)	0.99	0.99 (0.57, 1.71)	
[0.518, 23.636] (*n* = 1,805)	1.09	1.09 (0.59, 2.03)	1.08	1.08 (0.55, 2.11)	0.96	0.96 (0.48, 1.93)	
**C16:0**							
[–22.230, –2.123] (*n* = 1,806)	Ref						0.16
[–2.123, 0.161] (*n* = 1,806)	1.01	1.01 (0.73, 1.41)	0.92	0.92 (0.65, 1.29)	0.94	0.94 (0.66, 1.35)	
[0.161, 2.444] (*n* = 1,804)	1.35	1.35 (0.83, 2.21)	1.25	1.25 (0.76, 2.06)	1.26	1.26 (0.75, 2.12)	
[2.444, 46.1789] (*n* = 1,806)	1.36	1.36 (0.76, 2.42)	1.19	1.19 (0.66, 2.13)	1.20	1.20 (0.65, 2.22)	
**C18:0**							
[–10.703, –1.313] (*n* = 1,805)	Ref						0.005
[–1.313, –0.125] (*n* = 1,806)	1.56	1.56 (1.15, 2.11)	1.52	1.52 (1.12, 2.07)	1.48	1.48 (1.08, 2.04)	
[–0.125, 1.073] (*n* = 1,804)	1.33	1.33 (0.89, 1.97)	1.21	1.21 (0.81, 1.81)	1.17	1.17 (0.77, 1.78)	
[1.073, 17.2571] (*n* = 1,807)	1.32	1.32 (0.85, 2.05)	1.14	1.14 (0.73, 1.79)	1.09	1.09 (0.68, 1.73)	

^a^Multivariate logistic regression model adjusted for covariates (gender, age, race). ^b^Multivariate logistic regression models adjusted for covariates (gender, age, race, marital, ratio of family income to poverty levels, BMI, education levels, smoking consumption status, alcohol consumption status, PA). ^c^Multivariate logistic regression models adjusted for covariates (gender, age, race, marital, ratio of family income to poverty levels, education levels, BMI, smoking consumption status, alcohol consumption status, PA, protein, dietary fiber, total polyunsaturated fatty acids [total pfat]). ^d^Test for trend based on the variable containing a median value for each quartile.

In Tables 6, 7, female dietary intake of SFAs, C14: 0, C16: 0, C18: 0 fourth quartile, C14: 0 third quartile (OR [95% CI]) was as follows: (0.57 [0.34, 0.95]), (0.57 [0.34, 0.95]), (0.57 [0.34, 0.95]), (0.57 [0.34, 0.95]), (0.57 [0.34,0.95]), and (0.57 [0.34, 0.95]).

**TABLE 6:** Weighted OR (95% CI) for hypertension in quartiles of adjusted dietary saturated fatty acid intake stratified by gender in NHANES, 1999–2018.

Subgroup	Male (*n* = 3,778)	*P-value*	OR (95% CI)	Female (*n* = 3,444)	*P-value*	OR (95% CI)
**SFAs**						
	[–42.787, –5.854] (*n* = 945)	Ref		[–27.146, –3.582] (*n* = 861)	Ref	
	[–5.854, –0.566] (*n* = 944)	0.91	0.91 (0.50, 1.66)	[–3.582, 0.348] (*n* = 861)	1.10	1.10 (0.64, 1.92)
	[–0.566, 4.796] (*n* = 944)	0.83	0.83 (0.37, 1.87)	[0.348, 4.47] (*n* = 861)	1.04	1.04 (0.47, 2.30)
	[4.796, 116.405] (*n* = 945)	0.77	0.77 (0.29, 2.04)	[4.47, 49.052] (*n* = 861)	1.74	1.74 (0.61, 4.91)
**C4:0**						
	[–1.566, –0.328] (*n* = 948)	Ref		[–1.171, –0.195] (*n* = 865)	Ref	
	[–0.328, –0.123] (*n* = 939)	0.85	0.85 (0.48, 1.49)	[–0.195, –0.041] (*n* = 854)	0.69	0.69 (0.41, 1.16)
	[–0.123, 0.142] (*n* = 946)	1.07	1.07 (0.57, 2.01)	[–0.041, 0.157] (*n* = 864)	0.62	0.62 (0.32, 1.23)
	[0.142, 7.978] (*n* = 945)	1.80	1.80 (0.77, 4.22)	[0.157, 2.574] (*n* = 861)	0.69	0.69 (0.32, 1.48)
**C6:0**						
	[–0.874, –0.182] (*n* = 946)	Ref		[–0.647, –0.109] (*n* = 862)	Ref	
	[–0.182, –0.065] (*n* = 951)	0.79	0.79 (0.45, 1.40)	[–0.109, –0.021] (*n* = 863)	1.13	1.13 (0.66, 1.93)
	[–0.065, 0.082] (*n* = 936)	0.74	0.74 (0.36, 1.52)	[–0.021, 0.093] (*n* = 858)	1.55	1.55 (0.76, 3.16)
	[0.082, 4.301] (*n* = 945)	0.52	0.52 (0.23, 1.19)	[0.093, 1.424] (*n* = 861)	1.18	1.18 (0.50, 2.80)
**C8:0**						
	[–0.681, –0.134] (*n* = 941)	Ref		[–0.472, –0.077] (*n* = 859)	Ref	
	[–0.134, –0.046] (*n* = 947)	1.14	1.14 (0.62, 2.10)	[–0.077, –0.011] (*n* = 862)	1.33	1.33 (0.78, 2.28)
	[–0.046, 0.065] (*n* = 947)	1.51	1.51 (0.67, 3.41)	[–0.011, 0.081] (*n* = 859)	1.57	1.57 (0.67, 3.66)
	[0.065, 3.478] (*n* = 943)	1.38	1.38 (0.61, 3.14)	[0.081, 3.227] (*n* = 864)	1.04	1.04 (0.37, 2.91)
**C10:0**						
	[–1.291, –0.238] (*n* = 952)	Ref		[–0.896, –0.133] (*n* = 863)	Ref	
	[–0.238, –0.07] (*n* = 938)	0.79	0.79 (0.41, 1.54)	[–0.133, –0.007] (*n* = 857)	1.13	1.13 (0.62, 2.06)
	[–0.07, 0.143] (*n* = 942)	0.59	0.59 (0.26, 1.35)	[–0.007, 0.16] (*n* = 862)	0.89	0.89 (0.37, 2.12)
	[0.143, 5.596] (*n* = 946)	0.62	0.62 (0.20, 1.91)	[0.16, 3.288] (*n* = 862)	0.80	0.80 (0.30, 2.16)
**C12:0**						
	[–1.916, –0.394] (*n* = 945)	Ref		[–1.3798, –0.236] (*n* = 864)	Ref	
	[–0.394, –0.163] (*n* = 945)	1.28	1.28 (0.76, 2.16)	[–0.236, –0.055] (*n* = 861)	0.77	0.77 (0.47, 1.27)
	[–0.163, 0.166] (*n* = 943)	0.81	0.81 (0.46, 1.42)	[–0.055, 0.242] (*n* = 857)	0.62	0.62 (0.34, 1.13)
	[0.166, 22.855] (*n* = 945)	1.30	1.30 (0.77, 2.20)	[0.242, 20.7318] (*n* = 862)	0.69	0.69 (0.35, 1.35)
**C14:0**						
	[–5.382, –1.018] (*n* = 945)	Ref		[–4.3608, –0.638] (*n* = 861)	Ref	
	[–1.018, –0.314] (*n* = 944)	1.10	1.10 (0.58, 2.08)	[–0.638, –0.111] (*n* = 861)	0.90	0.90 (0.51, 1.61)
	[–0.314, 0.497] (*n* = 944)	1.15	1.15 (0.51, 2.60)	[–0.111, 0.529] (*n* = 862)	1.16	1.16 (0.54, 2.48)
	[0.497, 23.636] (*n* = 945)	0.96	0.96 (0.37, 2.52)	[0.529, 8.976] (*n* = 860)	1.06	1.06 (0.39, 2.88)
**C16:0**						
	[–22.230, –2.623] (*n* = 943)	Ref		[–14.070, –1.608] (*n* = 861)	Ref	
	[–2.623, –0.01] (*n* = 946)	0.75	0.75 (0.48, 1.17)	[–1.608, 0.306] (*n* = 861)	1.55	1.55 (0.96, 2.50)
	[–0.01, 2.654] (*n* = 945)	1.07	1.07 (0.58, 1.96)	[0.306, 2.267] (*n* = 861)	1.68	1.68 (0.88, 3.21)
	[2.654, 46.178] (*n* = 944)	0.94	0.94 (0.43, 2.03)	[2.267, 18.082] (*n* = 861)	1.40	1.40 (0.59, 3.32)
**C18:0**						
	[–10.703, –1.635] (*n* = 945)	Ref		[–7.027, –1.031] (*n* = 861)	Ref	
	[–1.635, –0.217] (*n* = 944)	1.75	1.75 (1.11, 2.76)	[–1.031, –0.057] (*n* = 861)	0.89	0.89 (0.59, 1.35)
	[–0.217, 1.256] (*n* = 944)	1.19	1.19 (0.67, 2.12)	[–0.057, 0.951] (*n* = 861)	0.97	0.97 (0.58, 1.64)
	[1.256, 17.257] (*n* = 945)	1.10	1.10 (0.58, 2.10)	[0.951, 12.788] (*n* = 861)	1.08	1.08 (0.57, 2.06)

Multivariate logistic regression models adjusted for covariates (age, race, marital, ratio of family income to poverty levels, BMI, education levels, smoking consumption status, alcohol consumption status, PA, protein, dietary fiber, total polyunsaturated fatty acids [total pfat]).

**TABLE 7:** Weighted OR (95% CI) for hypertension in quartiles of adjusted dietary saturated fatty acid intake stratified by age in NHANES, 1999–2018.

Subgroup	Age in 20–44 years (*N* = 4,840)	*P-value*	OR (95% CI)	Age in 45–64 years (*N* = 1,697)	*P-value*	OR (95% CI)	Age in ≥ 65 years (*N* = 685)	*P-value*	OR (95% CI)
**SFAs**									
	[–42.787, –4.705] (*n* = 1210)	Ref		[–31.396, –4.784] (*n* = 424)	Ref		[–26.892, –4.136] (*n* = 172)	Ref	
	[–4.705, –0.098] (*n* = 1209)	0.59	0.59 (0.32, 1.10)	[–4.784, –0.246] (*n* = 425)	1.00	1.00 (0.52, 1.91)	[–4.136, 0.008] (*n* = 171)	0.42	0.42 (0.11, 1.56)
	[–0.098, 4.741] (*n* = 1211)	0.46	0.46 (0.20, 1.03)	[–0.246, 4.207] (*n* = 424)	0.94	0.94 (0.37, 2.36)	[0.008, 4.438] (*n* = 171)	0.64	0.64 (0.13, 3.10)
	[4.741, 116.405] (*n* = 1210)	0.65	0.65 (0.26, 1.63)	[4.207, 40.582] (*n* = 424)	1.32	1.32 (0.41, 4.18)	[4.438, 34.101] (*n* = 171)	0.74	0.74 (0.10, 5.63)
**C4:0**									
	[–1.566, –0.259] (*n* = 1208)	Ref		[–1.242, –0.272] (*n* = 425)	Ref		[–1.070, –0.22] (*n* = 171)	Ref	
	[–0.259, –0.071] (*n* = 1207)	0.71	0.71 (0.47, 1.07)	[–0.272, –0.097] (*n* = 423)	0.76	0.76 (0.42, 1.38)	[–0.22, –0.061] (*n* = 172)	0.57	0.57 (0.20, 1.61)
	[–0.071, 0.157] (*n* = 1215)	0.66	0.66 (0.39, 1.12)	[–0.097, 0.107] (*n* = 424)	0.98	0.98 (0.47, 2.03)	[–0.061, 0.179] (*n* = 171)	0.39	0.39 (0.08, 1.80)
	[0.157, 7.978] (*n* = 1210)	1.20	1.20 (0.60, 2.41)	[0.107, 2.832] (*n* = 425)	0.81	0.81 (0.33, 2.01)	[0.179, 1.730] (*n* = 171)	0.26	0.26 (0.04, 1.47)
**C6:0**									
	[–0.874, –0.146] (*n* = 1211)	Ref		[–0.669, –0.151] (*n* = 422)	Ref		[–0.622, –0.114] (*n* = 171)	Ref	
	[–0.146, –0.04] (*n* = 1212)	0.60	0.60 (0.30, 1.17)	[–0.151, –0.05] (*n* = 424)	0.91	0.91 (0.48, 1.71)	(–0.114, –0.029] (*n* = 171)	3.49	3.49 (0.64, 19.01)
	[–0.04, 0.088] (*n* = 1205)	0.77	0.77 (0.35, 1.68)	[–0.05, 0.07] (*n* = 427)	0.77	0.77 (0.35, 1.70)	[–0.029, 0.115] (*n* = 172)	3.13	3.13 (0.48, 20.30)
	[0.088, 4.301] (*n* = 1212)	0.41	0.41 (0.17, 0.97)	[0.07, 1.830] (*n* = 424)	0.81	0.81 (0.31, 2.09)	[0.115, 1.042] (*n* = 171)	7.45	7.45 (0.78, 71.34)
**C8:0**									
	[–0.681, –0.108] (*n* = 1215)	Ref		[–0.545, –0.105] (*n* = 425)	Ref		[–0.472, –0.088] (*n* = 173)	Ref	
	[–0.108, –0.024] (*n* = 1207)	1.58	1.58 (0.81, 3.12)	[–0.105, –0.033] (*n* = 423)	0.76	0.76 (0.40, 1.47)	[–0.088, –0.023] (*n* = 167)	1.14	1.14 (0.35, 3.74)
	[–0.024, 0.077] (*n* = 1209)	2.33	2.33 (0.96, 5.66)	[–0.033, 0.066] (*n* = 426)	1.09	1.09 (0.48, 2.46)	[–0.023, 0.085] (*n* = 175)	2.61	2.61 (0.66, 10.40)
	[0.077, 3.325] (*n* = 1209)	2.18	2.18 (0.86,5.53)	[0.066, 3.478] (*n* = 423)	0.97	0.97 (0.32, 2.90)	[0.085, 1.82] (*n* = 170)	1.78	1.78 (0.37, 8.55)
**C10:0**									
	[–1.291, –0.186] (*n* = 1214)	Ref		[–0.948, –0.188] (*n* = 425)	Ref		[–0.8966, –0.153] (*n* = 171)	Ref	
	[–0.186, –0.031] (*n* = 1210)	1.02	1.02 (0.52, 2.00)	[–0.188, –0.047] (*n* = 423)	1.70	1.70 (0.86, 3.39)	[–0.153, –0.034] (*n* = 173)	0.68	0.68 (0.13, 3.61)
	[–0.031, 0.158] (*n* = 1205)	0.91	0.91 (0.38, 2.21)	[–0.047, 0.129] (*n* = 424)	1.59	1.59 (0.61, 4.13)	[–0.034, 0.159] (*n* = 169)	0.29	0.29 (0.05, 1.66)
	[0.158, 5.596] (*n* = 1211)	0.98	0.98 (0.34, 2.89)	[0.129, 2.529] (*n* = 425)	1.26	1.26 (0.35, 4.56)	[0.159, 1.979] (*n* = 172)	0.15	0.15 (0.02, 1.17)
**C12:0**									
	[–1.916, –0.325] (*n* = 1208)	Ref		[–1.564, –0.314] (*n* = 425)	Ref		[–1.379, –0.257] (*n* = 172)	Ref	
	[–0.325, –0.105] (*n* = 1209)	1.28	1.28 (0.83, 1.96)	[–0.314, –0.106] (*n* = 424)	0.70	0.70 (0.41, 1.22)	[–0.257, –0.088] (*n* = 169)	0.81	0.81 (0.36, 1.84)
	[–0.105, 0.209] (*n* = 1211)	0.89	0.89 (0.56, 1.40)	[–0.106, 0.224] (*n* = 422)	0.71	0.71 (0.39, 1.28)	[–0.088, 0.183] (*n* = 173)	0.57	0.57 (0.25, 1.34)
	[0.209, 20.731] (*n* = 1212)	1.00	1.00 (0.61, 1.65)	[0.224, 22.855] (*n* = 426)	0.56	0.56 (0.28, 1.13)	[0.183, 9.856] (*n* = 171)	2.01	2.01 (0.74, 5.45)
**C14:0**									
	[–5.382, –0.811] (*n* = 1211)	Ref		[–4.442, –0.853] (*n* = 425)	Ref		[–4.360, –0.739] (*n* = 172)	Ref	
	[–0.811, –0.175] (*n* = 1210)	0.88	0.88 (0.52, 1.51)	[–0.853, –0.262] (*n* = 424)	1.24	1.24 (0.64, 2.39)	[–0.739, –0.235] (*n* = 170)	1.03	1.03 (0.37, 2.85)
	[–0.175, 0.549] (*n* = 1209)	0.99	0.99 (0.46, 2.10)	[–0.262, 0.415] (*n* = 424)	1.24	1.24 (0.49, 3.13)	[–0.235, 0.517] (*n* = 171)	2.35	2.35 (0.58, 9.57)
	[0.549, 23.636] (*n* = 1210)	0.85	0.85 (0.31, 2.31)	[0.415, 7.308] (*n* = 424)	1.21	1.21 (0.35, 4.15)	[0.517, 5.731] (*n* = 172)	1.51	1.51 (0.29, 7.82)
**C16:0**									
	[–22.230, –2.132] (*n* = 1210)	Ref		[–16.503, –2.206] (*n* = 425)	Ref		[–13.304, –1.8] (*n* = 171)	Ref	
	[–2.132, 0.177] (*n* = 1210)	1.26	1.26 (0.75, 2.10)	[–2.206, 0.108] (*n* = 423)	0.67	0.67 (0.38, 1.18)	[–1.8, 0.13] (*n* = 171)	1.81	1.81 (0.68, 4.85)
	[0.177, 2.542] (*n* = 1210)	2.51	2.51 (1.31, 4.80)	[0.108, 2.27] (*n* = 425)	0.94	0.94 (0.41, 2.14)	[0.13, 2.235] (*n* = 171)	0.95	0.95 (0.28, 3.30)
	[2.542, 46.178] (*n* = 1210)	2.18	2.18 (0.94, 5.02)	[2.27, 18.574] (*n* = 424)	0.64	0.64 (0.24, 1.70)	[2.235, 17.061] (*n* = 172)	0.32	0.32 (0.07, 1.50)
**C18:0**									
	[–10.703, –1.349] (*n* = 1210)	Ref		[–8.652, –1.343] (*n* = 424)	Ref		[–6.807, –1.021] (*n* = 172)	Ref	
	[–1.349, –0.144] (*n* = 1209)	1.32	1.32 (0.87, 2.01)	[–1.343, –0.114] (*n* = 424)	2.17	2.17 (1.23, 3.83)	[–1.021, –0.049] (*n* = 170)	1.37	1.37 (0.60, 3.13)
	[–0.144, 1.063] (*n* = 1211)	1.01	1.01 (0.58, 1.74)	[–0.114, 1.098] (*n* = 424)	1.61	1.61 (0.79, 3.30)	[–0.049, 1.086] (*n* = 171)	1.16	1.16 (0.42, 3.20)
	[1.063, 17.257] (*n* = 1210)	0.86	0.86 (0.47, 1.59)	[1.098, 9.350] (*n* = 425)	1.59	1.59 (0.74, 3.41)	[1.086, 9.016] (*n* = 172)	1.87	1.87 (0.57, 6.18)

Multivariate logistic regression models adjusted for covariates (gender, race, marital, ratio of family income to poverty levels, BMI, education levels, smoking consumption status, alcohol consumption status, PA, protein, dietary fiber, total polyunsaturated fatty acids [total pfat]).

In Tables 8, 9, among (45–64, years) respondents, the (OR [95% CI]) of the second quartile of C6:0 and C14:0 were (1.67 [1.00, 2.78]) and (1.77 [1.05, 3.00]), respectively. Among (≥65, years) respondents with dietary intake of SFAs, the (OR [95% CI]) of the C4:0, C14:0, C16:0 fourth quartile, and C12:0 third quartile were (0.42 [0.21, 0.86]), (0.46 [0.22, 0.95]), (0.39 [0.18, 0.85]), (0.38 [0.17, 0.84]), and (0.45 [0.20, 0.99]), respectively.

**TABLE 8:** OR (95% CI) for hypertension in quartiles of adjusted intake of dietary saturated fatty acids and their subtypes interacted with sex in NHANES, 1999–2018.

Subgroups	*P*–*value*	OR (95% CI)
(SFAs) [–42.786, –4.638]: Women	Ref	
(SFAs) [–4.638, –0.128]: Women	0.21	1.33 (0.85, 2.06)
(SFAs) [–0.128, 4.571]: Women	0.41	1.25 (0.73, 2.12)
(SFAs) [4.571, 116.405]: Women	0.01	2.07 (1.23, 3.49)
(C4:0) [–1.565, –0.259]: Women	Ref	
(C4:0) [–0.259, –0.077]: Women	0.16	1.42 (0.87, 2.31)
(C4:0) [–0.077, 0.15]: Women	0.20	1.37 (0.84, 2.24)
(C4:0) [0.15, 7.975]: Women	0.26	1.34 (0.81, 2.21)
(C6:0) [–0.873, –0.145]: Women	Ref	
(C6:0) [–0.145, –0.041]: Women	0.46	1.18 (0.76, 1.85)
(C6:0) [–0.041, 0.086]: Women	0.10	1.54 (0.92, 2.59)
(C6:0) [0.086, 4.301]: Women	0.25	1.34 (0.82, 2.20)
(C8:0) [–0.681, –0.106]: Women	Ref	
(C8:0) [–0.106, –0.026]: Women	0.94	1.02 (0.64, 1.63)
(C8:0) [–0.026, 0.074]: Women	0.23	1.37 (0.81, 2.31)
(C8:0) [0.074, 3.478]: Women	0.55	1.16 (0.71, 1.89)
(C10:0) [–1.291, –0.183]: Women	Ref	
(C10:0) [–0.183, –0.035]: Women	0.28	1.29 (0.81, 2.07)
(C10:0) [–0.035, 0.15]: Women	0.17	1.43 (0.86, 2.40)
(C10:0) [0.15, 5.596]: Women	0.32	1.27 (0.79, 2.02)
(C12:0) [–1.916, –0.315]: Women	Ref	
(C12:0) [–0.315, –0.103]: Women	0.79	1.07 (0.67, 1.71)
(C12:0) [–0.103, 0.209]: Women	0.46	1.21 (0.72, 2.04)
(C12:0) [0.209, 22.855]: Women	0.41	1.22 (0.76, 1.98)
(C14:0) [–5.382, –0.819]: Women	Ref	
(C14:0) [–0.819, –0.2]: Women	0.07	1.53 (0.97, 2.41)
(C14:0) [–0.2, 0.518]: Women	0.04	1.72 (1.04, 2.85)
(C14:0) [0.518, 23.636]: Women	0.04	1.73 (1.02, 2.95)
(C16:0) [–22.230, –2.123]: Women	Ref	
(C16:0) [–2.123, 0.161]: Women	0.32	1.27 (0.79, 2.06)
(C16:0) [0.161, 2.444]: Women	0.32	1.31 (0.76, 2.25)
(C16:0) [2.444, 46.178]: Women	0.01	1.94 (1.16, 3.24)
(C18:0) [–10.703, –1.313]: Women	Ref	
(C18:0) [–1.313, –0.125]: Women	0.33	0.78 (0.46, 1.30)
(C18:0) [–0.125, 1.073]: Women	0.24	1.38 (0.81, 2.37)
(C18:0) [1.073, 17.257]: Women	0.03	1.79 (1.06, 3.03)

Multivariate logistic regression models adjusted for covariates (age, race, marital, ratio of family income to poverty levels, BMI, education levels, smoking consumption status, alcohol consumption status, PA, protein, dietary fiber, total polyunsaturated fatty acids [total pfat]).

**TABLE 9:** OR (95% CI) for hypertension in quartiles of adjusted intake of dietary saturated fatty acids and their subtypes interacted with age in NHANES, 1999–2018.

Subgroups	*P-value*	OR (95% CI)	Subgroups	*P-value*	OR (95% CI)
(SFAs) [–42.786, –4.638]: (45–64, years)	Ref		(SFAs) ([–42.786, –4.638]: (≥65, years)	Ref	
(SFAs) [–4.638, –0.128]: (45–64, years)	0.14	1.56 (0.86, 2.81)	(SFAs) [–4.638, –0.128]: (≥65, years)	0.81	0.91 (0.41, 1.99)
(SFAs) [–0.128, 4.571]: (45–64, years)	0.26	1.38 (0.78, 2.42)	(SFAs) [–0.128, 4.571]: (≥65, years)	0.74	0.88 (0.42, 1.88)
(SFAs) [4.571, 116.405]: (45–64, years)	0.98	0.99 (0.57, 1.73)	(SFAs) [4.571, 116.405]: (≥65, years)	0.02	0.42 (0.21, 0.86)
(C4:0) [–1.565, –0.259]: (45–64, years)	Ref		(C4:0) [–1.565, –0.259]: (≥65, years)	Ref	
(C4:0) [–0.259, –0.077]: (45–64, years)	0.18	1.42 (0.85, 2.39)	(C4:0) [–0.259, –0.077]: (≥65, years)	0.70	1.18 (0.52, 2.68)
(C4:0) [–0.077, 0.15]: (45–64, years)	0.15	1.51 (0.87, 2.62)	(C4:0) [–0.077, 0.15]: (≥65, years)	0.88	0.94 (0.45, 1.99)
(C4:0) [0.15, 7.978]: (45–64, years)	0.70	0.91 (0.54, 1.51)	(C4:0) [0.15, 7.978]: (≥65, years)	0.04	0.46 (0.22, 0.95)
(C6:0) [–0.873, –0.145]: (45–64, years)	Ref		(C6:0) [–0.873, –0.145]: (≥65, years)	Ref	
(C6:0) [–0.145, –0.041]: (45–64, years)	0.05	1.67 (1.00, 2.78)	(C6:0) [–0.145, –0.041]: (≥65, years)	0.41	1.36 (0.65, 2.84)
(C6:0) [–0.041, 0.086]: (45–64, years)	0.45	1.23 (0.71, 2.13)	(C6:0) [–0.041, 0.086]: (≥65, years)	0.13	0.53 (0.23, 1.20)
(C6:0) [0.086, 4.301]: (45–64, years)	0.76	1.08 (0.65, 1.79)	(C6:0) [0.086, 4.301]: (≥65, years)	0.14	0.54 (0.24, 1.21)
(C8:0) [–0.681, –0.106]: (45–64, years)	Ref		(C8:0) [–0.681, –0.106]: (≥65, years)	Ref	
(C8:0) [–0.106, –0.026]: (45–64, years)	0.85	0.95 (0.56, 1.61)	(C8:0) [–0.106, –0.026]: (≥65, years)	0.87	0.94 (0.43, 2.03)
(C8:0) [–0.026, 0.074]: (45–64, years)	0.78	0.92 (0.52, 1.63)	(C8:0) [–0.026, 0.074]: (≥65, years)	0.40	0.71 (0.32, 1.59)
(C8:0) [0.074, 3.478]: (45–64, years)	0.34	0.79 (0.48, 1.29)	(C8:0) [0.074, 3.478]: (≥65, years)	0.20	0.61 (0.29, 1.31)
(C10:0) [–1.291, –0.183]: (45–64, years)	Ref		(C10:0) [–1.291, –0.183]: (≥65, years)	Ref	
(C10:0) [–0.183, –0.035]: (45–64, years)	0.34	1.27 (0.78, 2.07)	(C10:0) [–0.183, –0.035]: (≥65, years)	0.43	1.33 (0.65, 2.71)
(C10:0) [–0.035, 0.15]: (45–64, years)	0.53	1.20 (0.67, 2.13)	(C10:0) [–0.035, 0.15]: (≥65, years)	0.37	0.71 (0.33, 1.53)
(C10:0) [0.15, 5.596]: (45–64, years)	0.69	0.90 (0.55, 1.50)	(C10:0) [0.15, 5.596]: (≥65, years)	0.08	0.51 (0.24, 1.08)
(C12:0) [–1.916, –0.315]: (45–64, years)	Ref		(C12:0) [–1.916, –0.315]: (≥65, years)	Ref	
(C12:0) [–0.315, –0.103]: (45–64, years)	0.34	0.78 (0.47, 1.30)	(C12:0) [–0.315, –0.103]: (≥65, years)	0.43	0.76 (0.38, 1.50)
(C12:0) [–0.103, 0.209]: (45–64, years)	0.85	1.06 (0.59, 1.90)	(C12:0) [–0.103, 0.209]: (≥65, years)	0.05	0.45 (0.20, 0.99)
(C12:0) [0.209, 22.855]: (45–64, years)	0.15	0.69 (0.41, 1.15)	(C12:0) [0.209, 22.855]: (≥65, years)	0.33	0.69 (0.33, 1.45)
(C14:0) [–5.382, –0.819]: (45–64, years)	Ref		(C14:0) [–5.382, –0.819]: (≥65, years)	Ref	
(C14:0) [–0.819, –0.2]: (45–64, years)	0.03	1.77 (1.05, 3.00)	(C14:0) [–0.819, –0.2]: (≥65, years)	0.57	0.80 (0.37, 1.73)
(C14:0) [–0.2, 0.518]: (45–64, years)	0.28	1.39 (0.76, 2.52)	(C14:0) [–0.2, 0.518]: (≥65, years)	0.44	0.72 (0.32, 1.65)
(C14:0) [0.518, 23.636]: (45–64, years)	0.69	1.11 (0.65, 1.92)	(C14:0) [0.518, 23.636]: (≥65, years)	0.02	0.39 (0.18, 0.85)
(C16:0) [–22.230, –2.123]: (45–64, years)	Ref		(C16:0) [–22.230, –2.123]: (≥65, years)	Ref	
(C16:0) [–2.123, 0.161]: (45–64, years)	0.49	1.21 (0.71, 2.06)	(C16:0) [–2.123, 0.161]: (≥65, years)	0.9	1.05 (0.47, 2.32)
(C16:0) [0.161, 2.444]: (45–64, years)	0.97	0.99 (0.58, 1.70)	(C16:0) [0.161, 2.444]: (≥65, years)	0.38	0.69 (0.31, 1.57)
(C16:0) [2.444, 46.178]: (45–64, years)	0.44	0.80 (0.45, 1.42)	(C16:0) [2.444, 46.178]: (≥65, years)	0.02	0.38 (0.17, 0.84)
(C18:0) [–10.703, –1.313]: (45–64, years)	Ref		(C18:0) [–10.703, –1.313]: (≥65, years)	Ref	
(C18:0) [–1.313, –0.125]: (45–64, years)	0.16	1.51 (0.84, 2.71)	(C18:0) [–1.313, –0.125]: (≥65, years)	0.69	0.87 (0.43, 1.74)
(C18:0) [–0.125, 1.073]: (45–64, years)	0.57	1.19 (0.65, 2.17)	(C18:0) [–0.125, 1.073]: (≥65, years)	0.30	0.68 (0.32, 1.43)
(C18:0) [1.073, 17.257]: (45–64, years)	0.57	1.19 (0.65, 2.18)	(C18:0) [1.073, 17.257]: (≥65, years)	0.11	0.55 (0.26, 1.16)

Multivariate logistic regression models adjusted for covariates (gender, race, marital, ratio of family income to poverty levels, BMI, education levels, smoking consumption status, alcohol consumption status, PA, protein, dietary fiber, total polyunsaturated fatty acids [total pfat]).

## Discussion

The aim of this study was to investigate the relationship between SFAs and their subtypes and hypertension. The main findings were that there was no significant correlation between dietary intake of SFAs and their subtypes on hypertension in the logistic regression models (I, II, and III). Hypertension may be more likely to occur when the dietary intake of female respondents is in the fourth quartile of SFAs, the third and fourth quartiles of C14:0, the fourth quartile of C16:0, and the fourth quartile of C18:0. Hypertension may be more likely to occur when dietary intake in middle-aged adults (45–64, years) is in the second quartile of C6:0 and C14:0. Hypertension may be more likely to occur when dietary intake in older adults (≥65, years) is in the fourth quartile of SFAs, fourth quartile of C4:0, third and fourth quartiles of C12:0, fourth quartile of C14:0, and fourth quartile of C16:0.

The study found no significant association between dietary intake of fatty acids and their subtypes and hypertension in overall respondents, and the results of a community cohort study (23) and a meta-analysis (24) were similar to our results. Dietary intake of SFAs is considered a risk factor in the cardiovascular field, particularly high intakes of red meat and high-fat dairy products, which are the major sources of SFAs in the diet. The American Heart Association recommends and limits a daily intake of < 7% SFAs (8). However, some studies have found that intake of SFAs can lead to a lower risk of hypertension (25). Short- to medium-chain SFAs (C4:0–C10:0) were not significantly associated with the risk of coronary heart disease (26), and high intakes of long-chain fatty acids C14:0 and C16:0 were negatively associated with the risk of developing hypertension in older adults. Moreover, differences in chain length account for their differential effects on the risk of coronary heart disease (7), prompting further discussion of the effect of SFA subtypes on hypertension effects.

In female respondents, we found an interaction between SFAs, C14:0, C16:0, and C18:0 and hypertension. When dietary intake was high, SFAs may be a risk factor for hypertension. The correlation between dietary intake of SFAs and their subtypes and hypertension has been less studied in previous studies. However, gender differences in hypertension are common in the cardiovascular field (11). A review reported that high levels of serum SFA were associated with hypertension in men but not in women; and the high prevalence of hypertension in older women was partly due to decreased ovarian estrogen production caused by elevated blood pressure after menopause (27). However, more evidence is needed to determine whether this result is associated with a dietary intake of SFAs. A previous prospective cohort study in women noted that C4:0, C6:0, C8:0, and C10:0 were not associated with the risk of developing coronary heart disease, but C14:0, C16:0, and C18:0 were associated with the risk of coronary heart disease (26). Moreover, the serum cholesterol elevating effect of dietary SFAs may be related to C14:0, C16:0, and C18:0 (28, 29), which is similar to our findings.

We found that age interacted with SFAs, C4:0, C6:0, C12:0, C14:0, and C16:0. Age is considered an important influencing factor for hypertension. In the middle-aged population, dietary intake of C6:0 and C14:0 may be a risk factor for hypertension. SFAs were strongly associated with hypertension in the elderly, and total SFA, C4:0, C12:0, C14:0, and C16:0 may be protective factors for hypertension. Our findings were consistent with those obtained by a study in Japan (7). That is, C12:0 increases the activity of superoxide dismutase, catalase, and glutathione peroxidase, improving antioxidant activity (30). C12:0 improves vascular endothelial function and delays vascular aging, which is associated with impaired pressure-sensing reflex sensitivity and the development of hypertension (31). Reactive oxygen species contribute to the reduction of pressure reflex sensitivity (32, 33). Therefore, the observed association between SFAs and their isoforms and hypertension may be attributed to their antioxidant activity, which ultimately exhibits a protective effect. The fatty acid composition of plasma cholesteryl esters was positively associated with the 6-year incidence of hypertension in the ARIC study, but the mean age of the respondents was <65 years (23), which may indicate that age differences in distribution led to differences in results.

Considering that the underlying mechanisms were not examined in this study, the differences in the results might be due to several possible reasons: first, there may be internal mechanisms for SFAs and their subtypes, and differences in the confounding factors adjusted by regression analysis may lead to differences in the results. Second, SFA intake was measured by using the FFQ, a self-reporting-dependent tool that may lead to errors. Third, the possibility of reverse causation cannot be ruled out because of observational studies. Fourth, Neyman bias may exist during the NHANES survey. Fifth, only two measurements of dietary intake were averaged. Although diabetes and hyperlipidemia were excluded, long-term dietary habits and whether dietary habits changed after disease diagnosis could not be detected.

## Conclusion

This study was based on the National Health and Nutrition Examination Survey of the US population, and no significant effect of total SFAs was found in the total respondents. Female respondents may need to be aware that higher dietary intake of saturated fatty acids (SFAs, C14:0, C16:0, C18:0) may be a risk factor for hypertension. Dietary intake of SFAs, C4:0, C6:0, C12:0, C14:0, and C16:0 may be a protective factor for hypertension when higher in older adults. Women and elderly respondents in the United States should be cautious in their intake of SFAs to actively prevent the development of hypertension.

## Data availability statement

The original contributions presented in this study are included in the article/supplementary material, further inquiries can be directed to the corresponding authors.

## Author contributions

RG, YG, and JQ had full access to all of the data in the study and take responsibility for the integrity of the data and the accuracy of the data analysis. RG and YG drafted the manuscript. QG, TLu, ZC, YL, KH, SX, RL, TLi, JX, and YC critically revised the manuscript for important intellectual content. RG contributed to the statistical analysis. ZZ obtained the funding. ZZ and YL supervised the manuscript. All authors contributed to the article and approved the submitted version.

## Footnotes

1
https://www.cdc.gov/nchs/nhanes/irba98.htm

2
https://wwwn.cdc.gov/Nchs/Nhanes/2017-2018/DSQTOT_J.htm
